# Nonlinear Creep Damage Constitutive Model of Concrete Based on Fractional Calculus Theory

**DOI:** 10.3390/ma12091505

**Published:** 2019-05-08

**Authors:** Cong Zhang, Zhende Zhu, Shu Zhu, Zhilei He, Duan Zhu, Jinzhong Liu, Songsong Meng

**Affiliations:** 1 Key Laboratory of Ministry of Education for Geomechanics and Embankment Engineering, Hohai University, Nanjing 210098, China; zhangcong_edu999@163.com (C.Z.); 13851886169@163.com (Z.Z.); zhushuwork2010@163.com (S.Z.); jzliu89@hhu.edu.cn (J.L.); Mengsongsong_edu@163.com (S.M.); 2 Jiangsu Research Center for Geotechnical Engineering Technology, Hohai University, Nanjing 210098, China; 3 Henan Province Key Laboratory of Rock and Soil Mechanics and Structural Engineering, North China University of Water Resources and Electric Power, Zhengzhou 450045, China; zlhenj@163.com

**Keywords:** concrete creep, fractional derivative, nonlinear, creep damage, constitutive model, sensitivity analysis

## Abstract

Concrete creep has become one of the major problems that threatens concrete structural development and construction. However, a reasonable and accurate calculation model for numerical analysis is the key to control and solve the creep deformation of concrete. To better describe the concrete nonlinear creep damage evolution rule, the visco-elasticity Plasticity Rheological Theory, Riemann Liouville Theory and Combined Model Theory are quoted, and the Able dashpot is used to reconstruct fractional-order soft-body composite elements to propose the expression of the stress-strain relationship of the elastomer, visco-elasticity plasticity body, and Viscoplasticity body, considering the evolution of the concrete compression damage process. A nonlinear creep damage constitutive model of concrete, based on fractional calculus theory, is conducted, and the parameters of the specific calculation method of the model are given. The influence of stress level σ, fractional order n and material parameter α on the concrete creep process is determined by a sensitivity analysis of the model parameters. The creep process and deformation amount of concrete in practical engineering can be effectively controlled by the results of the proposed sensitivity analysis. The research results can be used to provide guidance and reference for the safe construction of concrete engineering in actual practice.

## 1. Introduction

Concrete creep refers to the phenomenon in which concrete deformation increases with time under long-term external load [1]. Creep development increases stress loss in the concrete structure. It also redistributes the internal force of static and statically indeterminate structures, resulting in excessive structural deformation, a significant reduction in overall strength, and even a loss of bearing capacity. With the current wide range, high quality, and general use of concrete materials, concrete creep has become one of the major problems that threatens concrete structural development and the safety of concrete construction and long-term stability [2,3].

Creep strain has a linear relationship with applied stress in the case of low stress levels (less than 30% compressive strength). However, creep presents an unstable phenomenon as time and stress increase in the case of high stress levels (between 30% and 80% compressive strength); that is, it presents a nonlinear relationship, which is found in concrete creep theories and experimental research [4,5,6,7]. At present, many linear creep models have been proposed to predict concrete creep deformation, such as the CEB-FIP model [8], the EC2 model [9], the ACI209 model [10], the GL2000 model [11], etc. The B3 model is constructed by using the Humidity Diffusion Theory and Consolidation Theory, and the various material parameters are determined through the specific concrete tests of Bazant [12,13]. The B4 model is proposed based on the B3 model, considering the effects of ambient temperature and self-shrinkage described by Mija H. Hubler [14,15]. This calculation analysis method and the inverse analysis method were introduced to conduct a concrete creep-fatigue constitutive model, based on the application of continuous damage mechanics that was proposed by Farnam Ghasemzadeh [16] and Vitaliy M. Kindrachuk et al. [17]. However, concrete nonlinear creep is very complex because it can be regarded as a composite material. The basic Maxwell model is referenced to propose the linearized mathematical modeling of the geometric nonlinearity theory proposed by Grenacher and Thurlimann [18]. But, the accuracy of concrete structure creep deformation cannot be accurately described by the above approximate processing model. 

The nonlinear creep process of reinforced concrete columns was simulated considering the high stress conditions by Bažant and Tsubaki [19]. A simple numerical model was conducted to simplify the concrete nonlinear creep calculation process of Kwak and Kim [20]. Geometrical nonlinearity and concrete fracture development were described using a layered method, and slender reinforced concrete column creep deformation was predicted in the model presented by Kwak and Kim. And, a numerical model for analysis of reinforced and prestressed concrete plates and shells, including creep, shrinkage, and aging of concrete, already developed by Radnić and Matešan [21], can and the model can be applied for all levels of concrete stresses, while its use for ultimate stress levels was still not fully tested. For most multi-coefficient nonlinear creep models, the range of parameters can be further extended, and the time effectiveness and applicability of nonlinear creep have yet to be further investigated and verified for concrete linear and nonlinear creep. 

Some strain in creep is related to the corresponding instantaneous stress state, and also to the previous stress state, which is illustrated by a large number of concrete creep phenomena. The fractional derivative, based on time parameters, is an arbitrary order time differential operator, which can fully reflect the course of the function change over time [22,23]. Fractional Calculus Theory is widely used in the field of materials [24], and the Fractional Derivative was introduced by Papoulia, K.D. [25] to establish a Viscoelastic Rheological Model that reveal the viscoelastic behavioral and material mechanical response. Viscoelastic behavior and response are consistent with the experimental results. The visco-elasticity plasticity model, based on the fractional derivative, was established to improve the viscoelastic-plastic response characteristics of materials by Suzuki, J.L. et al. [26]. A high temperature concrete creep nonlinear thermal viscoelastic creep model based on the fractional derivative was proposed to better describe nonlinear creep under conditions of high stress and high temperature by Bouras, Y. et al. [27]. The concrete fractional order genetic aging model was conducted by Beltempo, Angela et al. [28] in the variable-order fractional order calculus framework. The semi-analytic expression of material parameters was determined by using fractional order calculus forms.

To better describe the concrete nonlinear creep damage evolution rule, Fractional Calculus Theory and Rheological Model Theory have been quoted to propose a non-linear creep damage constitutive model of concrete using Able dashpot to replace Newton dashpot and the concrete damage evolution rule. The rationality and applicability of the conducted constitutive work was verified. Finally, the calculation method and sensitivity analysis of the conducted model parameters provide a reference for safe construction and the long-term stable operation of concrete engineering.

## 2. Construction of Concrete Nonlinear Creep Damage Constitutive Model

### 2.1. Riemann Liouville Fractional Order Software Components

There are many fractional order calculus definitions, among which the creep characteristics of concrete are defined and described by Riemann Liouville theory [29,30]. This is how the Riemann Liouville fractional calculus of function $f\left(t\right)$ is defined: f(·) is set on the (0, +∞) continuum and can be accumulated on any subinterval of [0, +∞], for *t* > 0, *Re*(*n*) > 0.

(1) $$D^{-n}f(t)=\int_{0}^{t}\frac{{\left(t-\tau \right)}^{n-1}}{\Gamma (n)}f(\tau )d\tau$$

The Riemann Liouville fractional order differential form is defined as.

(2) $$D^{n}f(t)=\frac{d^{a}}{dt^{a}}\int_{0}^{t}\frac{f{\left(t-\tau \right)}^{a-n-1}}{\Gamma (a-n)}f(\tau )d\tau$$

where $\Gamma (n)=\int_{0}^{\infty }t^{n-1}e^{-t}dt$ is the gamma function, *n* (*n >* 0) is the fractional order, a(a−1≤n≤a) is a positive integer larger than *n*, and *d* is a differential operator. The ideal solid stress-strain relationship follows Hooke’s law.

(3) $$\sigma \left(t\right)=E\varepsilon \left(t\right)$$

The ideal fluid stress-strain relationship follows Newton’s viscous law.

(4) $$\sigma \left(t\right)=\eta \frac{d\varepsilon (t)}{dt}$$

where σ(t) is a function of stress versus time, ε(t) is a function of strain versus time, E is the elastic modulus, and t is time.

**Figure 1:**
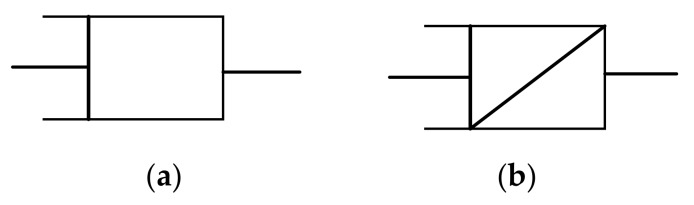
(**a**) Newton dashpot; (**b**) Able dashpot.

Ideal solids and ideal fluids are idealized models, which can only be used for approximate calculations in practical engineering. Concrete is a material between an ideal solid and an ideal fluid, and a software element [31] can be used to describe an intermediate material between a pure elastomer and a Newtonian fluid. Therefore, the concrete rheological properties can be described by combining a model with the Able dashpot instead of the Newton dashpot. The Newton dashpot and the Able dashpot are shown in Figure 1.

The constitutive equation based on the fractional derivative [32] is established as.

(5) $$\sigma \left(t\right)=\eta^{n}\frac{d^{n}\varepsilon (t)}{dt^{n}},0\leq n\leq 1$$

where the stress σ remains constant; that is, σ=const. This software element can describe the creep deformation. Fractional order integrals are performed on both sides of the Equation (5), by using the Riemann Liouville fractional order integral definition; that is.

(6) $$\varepsilon (t)=\frac{\sigma }{\eta^{n}}\frac{t^{n}}{\Gamma (1+n)},0\leq n\leq 1$$

### 2.2. Establishment and Solution of Fractional Order Creep Damage Model

In current research, the basic rheological mechanics models include elastomers, the viscoelastic body, the visco-elasticity plasticity body, the viscous body and the viscoplastic body. The rheological properties of materials can be described by the combination of strings and parallelism among them. The combined model has been widely used in the creep simulation research of asphalt, rock, polymer materials, concrete, and other materials because of its visualization and intuition capabilities [33,34,35].

**Figure 2:**
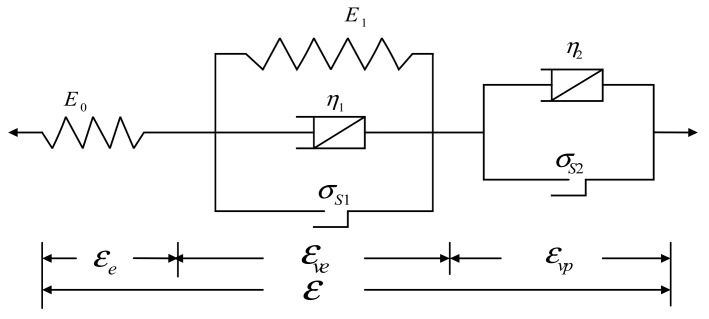
Creep Constitutive model.

The typical concrete compression and creep process consists of three stages: deceleration creep, steady creep, and accelerated creep. In the current study, the elastic-visco-elasticity plasticity-viscoplastic creep model (MSSB, Modified-Schofield-Scott- Blair) is used to describe the instantaneous and isovelocity creep of concrete. However, when concrete reaches the yield limit, the creep curve will exhibit accelerated unsteady characteristics. At this time, the MSSB model cannot describe the nonlinear creep characteristics of concrete, and it can only be studied by nonlinear theory. In this paper, the creep constitutive Equation based on the fractional derivative is constructed by using the Able dashpot, instead of the Newton dashpot in the MSSB model to accurately describe the three stages of deceleration creep, steady creep and accelerated creep. The constitutive model combination is shown in Figure 2.

To derive the constitutive equation, the main hypothesis conditions are given.

1. The small damage produced is neglected during the deceleration creep and steady creep stages, and only the damage caused by the accelerated creep stage is considered in the concrete indoor creep test.
2. The concrete damage evolution in the accelerated creep process is consistent with the change in the exponential function.

Based on the above constitutive models and assumptions, the stress-strain relationship between elastomers, the visco-elasticity plasticity body, and the viscoplastic body are derived as follows.

1. Stress-strain relationship of elastomer.
  (7) $$\varepsilon_{e}=\frac{\sigma }{E_{0}}$$
  where $E_{0}$ is the elastomer elastic modulus.
2. Stress-strain relationship of visco-elasticity plasticity body.The visco-elasticity plasticity body is represented in parallel with the elastomer element, the Able dashpot and the friction element. The stress on the friction element is expressed by $\sigma_{P1}$.
  (8) $$\sigma_{P1}=\left\{\begin{array}{ll} \sigma & \sigma <\sigma_{S1}\\ \sigma_{S1} & \sigma \geq \sigma_{S1} \end{array}\right.$$
  where σ is the total stress on the visco-elasticity plasticity body, and $\sigma_{S1}$ is the ultimate stress on the visco-elasticity plasticity body friction slider.

The total stress on the visco-elasticity plasticity body can be obtained from the combined model theory.

(9) $$\sigma =\sigma_{d1}+\sigma_{P1}$$

where $\sigma_{d1}$ is the stress on the Able dashpot.

When $\sigma \leq \sigma_{S1}$

(10) $$\sigma_{d1}=0,\varepsilon_{ve}=0$$

when $\sigma >\sigma_{S1}$.

(11) $$\sigma =\sigma_{S1}+E_{1}\varepsilon_{ve}+\eta_{1}{}^{n}\frac{d^{n}\varepsilon_{ve}}{dt^{n}}$$

where $E_{1}$ is the elastic modulus, and $\eta_{1}$ is the Able dashpot viscous coefficient.

Equation (11) can also be expressed as follows.

(12) $$\frac{\sigma -\sigma_{S1}}{\eta_{1}{}^{n}}=\frac{E_{1}}{\eta_{1}{}^{n}}\varepsilon_{ve}+\frac{d^{n}\varepsilon_{ve}}{dt^{n}}$$

Laplace transformation of Equation (12).

(13) $$E_{S}=\frac{\sigma -\sigma_{S1}}{\eta_{1}{}^{n}s}\frac{\eta_{1}{}^{n}}{s^{n}\eta_{1}{}^{n}+E_{1}}$$

Laplace inverse transformation of Equation (13).

(14) $$\varepsilon_{ve}=\frac{\sigma -\sigma_{S1}}{\eta_{1}{}^{n}}\sum_{k=0}^{\infty }\frac{{\left(-1\right)}^{k}{\left(E_{1}/\eta_{1}\right)}^{k}t^{n(1+k)}}{n(1+k)\Gamma \left[n(1+k)\right]}$$

where n is the order of the fractional calculus and Γ(·) is the gamma function.

(3) Stress-strain relationship of viscoplastic body. The viscoplastic body is expressed in parallel with the friction element by the Able dashpot. The stress $\sigma_{P2}$ on the friction element is expressed by the Able dashpot, which satisfies the following requirements.

(15) $$\sigma_{P2}=\left\{\begin{array}{l} \sigma \begin{array}{cc} & \sigma <\sigma_{S2} \end{array}\\ \sigma_{S2}\begin{array}{cc} & \sigma \geq \sigma_{S2} \end{array} \end{array}\right.$$

where σ is the total stress on the visco-elasticity plasticity body, and $\sigma_{S2}$ is the ultimate stress of the friction slider in the viscoplastic body. The total stress on the viscoplastic body can be obtained from the combination model theory.

(16) $$\sigma =\sigma_{d2}+\sigma_{P2}$$

where $\sigma_{d2}$ is the stress on the Able dashpot.

When $\sigma \leq \sigma_{S2}$

(17) $$\varepsilon_{vp}=0$$

when $\sigma >\sigma_{S2}$.

(18) $$\sigma =\sigma_{S2}+\eta_{2}{}^{n}\frac{d^{n}\varepsilon_{vp}}{dt^{n}}$$

where $\sigma_{S2}$ is the yield stress, and $\eta_{2}$ is the viscosity coefficient of the Able dashpot.

According to the theory of fractional calculus [32,36], the following can be obtained:

(19) $$\varepsilon_{vp}=\frac{\sigma -\sigma_{S2}}{\eta_{2}{}^{n}}\frac{t^{n}}{\Gamma (1+n)}$$

During the creep test process, especially in the accelerated creep stage, the creep rate of the concrete specimens increases continuously until failure, which is caused by the expansion of internal damage and crack development in the concrete. Therefore, the viscosity coefficient is no longer a fixed value, but rather is a variable that decreases with the increase in the degree of damage. The damage variable is a negative exponential function [37] that considers the influence of damage growth on the creep process, which can be expressed as.

(20) D=1−exp(−αt)

where D is the damage variable, and its value range is 1], which corresponds to the concrete failure state without damage, and the complete damage, respectively. α is a material parameter, which controls the damage evolution process and is related to the applied load. At this time, the viscous coefficient changes with the damage variable, which can be expressed as:

(21) $$\eta_{2}{}^{n}(t,D)=\eta_{2}{}^{n}(1-D)=\eta_{2}{}^{n}e^{-\alpha t}$$

then

(22) $$\varepsilon_{vp}=\frac{\sigma -\sigma_{S2}}{\eta_{2}{}^{n}e^{-\alpha t}}\frac{t^{n}}{\Gamma (1+n)}$$

Combining the above three parts, the strain and nonlinear creep damage constitutive model of concrete, based on fractional calculus theory, is obtained as

(23) $$\varepsilon =\left\{\begin{array}{l} \frac{\sigma }{E_{0}}+\frac{\sigma -\sigma_{S1}}{\eta_{1}{}^{n}}\sum_{k=0}^{\infty }\frac{{\left(-1\right)}^{k}{\left(E_{1}/\eta_{1}\right)}^{k}t^{n(1+k)}}{n(1+k)\Gamma \left[n(1+k)\right]}\begin{array}{ccc} & & \sigma_{S1}\leq \sigma \leq \sigma_{S2} \end{array}\\ \frac{\sigma }{E_{0}}+\frac{\sigma -\sigma_{S1}}{\eta_{1}{}^{n}}\sum_{k=0}^{\infty }\frac{{\left(-1\right)}^{k}{\left(E_{1}/\eta_{1}\right)}^{k}t^{n(1+k)}}{n(1+k)\Gamma \left[n(1+k)\right]}+\frac{\sigma -\sigma_{S2}}{\eta_{2}{}^{n}e^{-\alpha t}}\frac{t^{n}}{\Gamma (1+n)}\begin{array}{cc} & \begin{array}{cc} & \sigma >\sigma_{S2} \end{array} \end{array} \end{array}\right.$$

## 3. Determination of Concrete Nonlinear Creep Damage Constitutive Model Parameters

### 3.1. Determination of Parameter $E_{0}$

The parameter $E_{0}$ is the elastomer elastic modulus in the instantaneous state, which is calculated by the elastic strain section of the creep test at a certain stress level. The expression of parameter $E_{0}$ is as follows.

(24) $$E_{0}=\frac{\sigma_{0}}{\varepsilon_{0}}$$

where $\sigma_{0}$ is the initial stress, and $\varepsilon_{0}$ is the strain in the elastic stage.

### 3.2. Determination of Parameters $E_{1}$, $\sigma_{S1}$, and $\eta_{1}$

When the creep curve has an unloading curve, the three parameters $E_{1}$,$\sigma_{S1}$ and $\eta_{1}$ of the creep model can be calculated from the strain when the creep tends to be stable under a certain level of horizontal stress and under residual strain after unloading [38]. The expressions of $E_{1}$, $\sigma_{S1}$, $\eta_{1}$ are as follows.

(25) $$E_{1}=\frac{\sigma_{0}}{\varepsilon_{a}(\infty )+\varepsilon_{b}(\infty )},\sigma_{S1}=E_{1}\varepsilon_{b}(\infty ),\eta_{1}=-\frac{E_{1}t}{\ln(1-\varepsilon_{a}(t)/\varepsilon_{a}(\infty ))}$$

where $\varepsilon_{a}(\infty )$ is the strain when the creep tends to be stable under a certain stress loading. $\varepsilon_{b}(\infty )$ is a creep variable that cannot be recovered after unloading under a certain stress load. *t* is the creep time $\varepsilon_{a}(t)$ is the creep value corresponding to time *t*, where $\left(t,\varepsilon_{a}(t)\right)$ is any point in the creep viscoelastic stage. $\eta_{1}$ can select the average value of the calculation results from multiple data points.

A nonlinear regression analysis method [39] can be used to solve for parameters $E_{1}$, $\sigma_{S1}$**,** and $\eta_{1}$ when there is no unloading curve.

### 3.3. Determination of Parameters $\eta_{2}$ and $\sigma_{S2}$

The parameters $\eta_{2}$ and $\sigma_{S2}$ in the Viscoplasticity body can be obtained from the creep test curve rate in two groups when there is constant creep data in the experimental data. The expression is as follows.

(26) $${\dot{\varepsilon }}_{c}=\frac{\sigma_{1}-\sigma_{S1}}{\eta_{2}},{\dot{\varepsilon }}_{d}=\frac{\sigma_{2}-\sigma_{S2}}{\eta_{2}}$$

which are Calculated by Equation (26).

(27) $$\eta_{2}=\frac{\sigma_{2}-\sigma_{1}}{{\dot{\varepsilon }}_{d}-{\dot{\varepsilon }}_{c}},\sigma_{S2}=\frac{{\dot{\varepsilon }}_{d}\sigma_{2}-{\dot{\varepsilon }}_{c}\sigma_{1}}{{\dot{\varepsilon }}_{d}-{\dot{\varepsilon }}_{c}}$$

where $\sigma_{1}$ and $\sigma_{2}$ are two stress values of the creep test. ${\dot{\varepsilon }}_{c}$ and ${\dot{\varepsilon }}_{d}$ are creep rates at constant velocity under two stress levels. The concrete creep process will appear as third stage creep when the stress of the concrete is greater than its long-term strength. According to Equation (21), the relationship can be described by the exponential relationship. The viscosity coefficient decreases with the increase of time, and its variation law is related to the magnitude of the applied stress. At the same time, the greater the applied stress, the smaller the viscous coefficient. When the applied stress is large, the nonlinear regression analysis method can be used to solve for the parameters $\eta_{2}$ and $\sigma_{S2}$ due to the lack of two stress level isometric creep data.

## 4. Verification of Concrete Nonlinear Creep Damage Constitutive Model

### 4.1. Experimental Research on Concrete Creep under High Stress

**Figure 3:**
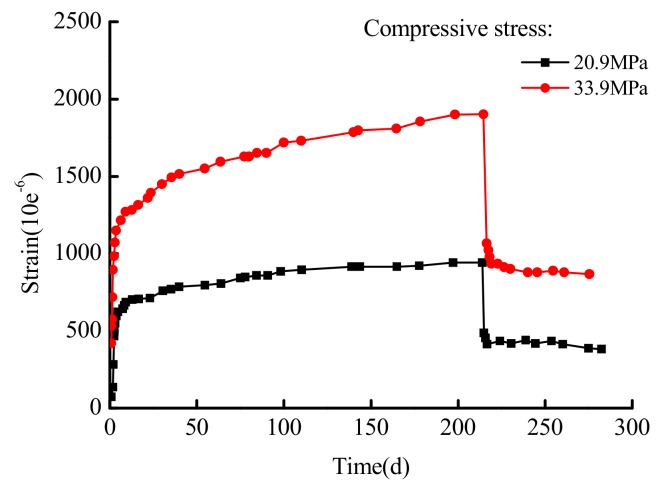
The curve of the concrete creep tests under different stress.

Uniaxial compressive nonlinear non-linear creep and residual strain tests of normal concrete with deep storage nuclear waste were carried out by Narintsoa Ranaivomanana, et al. [40]. The specimen size was 100 mm × 100 mm × 500 mm. A series of uniaxial compression nonlinear creep tests under different stresses and maintaining 20 °C were carried out. The stress was controlled at 30% (about 20.9 MPa) and 50% (about 33.9 MPa). The cement class was CEM I 52.5R(CEM I is the abbreviation for Portland cement.), the modulus of elasticity was 41925 MPa, the modulus of elasticity was 4295 MPa, and the average density was 2410 kg/m^3^. The experimental curves are shown in Figure 3.

**Figure 4:**
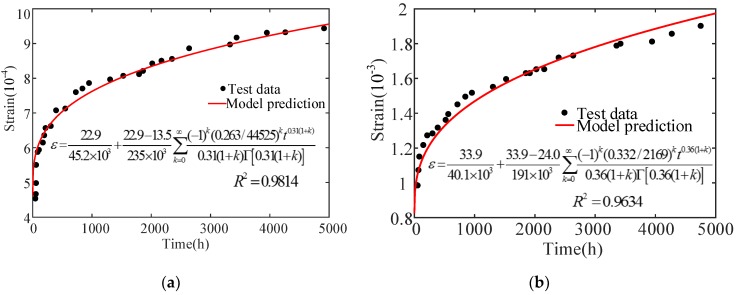
The fitting curve of the test curve and the model at (**a**) 20.9 MPa and (**b**) 33.9 MPa).

As shown in Figure 3, the concrete specimen nonlinear creep process can be divided into two stages under an uniaxial compression of stress levels of 20.9 MPa and 33.9 MPa: (1) the deceleration creep stage, with increasing strain and decreasing strain rate, and (2) the isovelocity creep stage with constant strain rate. The experimental curve characteristics can be described by using the elastomer and viscoelastic body in the model. Now, the parameters $E_{0}$, $E_{1}$, $\sigma_{S1}$, $\eta_{1}$, and n need to be solved. The numerical fitting analysis of Narintsoa Ranaivomanana, et al.’s [40] experimental results is carried out using the conducted model. The calculated model parameters are shown in Table 1, and the fitting results are shown in Figure 4. Where ‘-’ means that there is no such value. Accelerate creep has not occurred when the axial pressure is 20.9 MPa and 33.9 MPa, the concrete creep process consists of deceleration and isometric creep.

Narintsoa Ranaivomanana et al.’s [40] experimental curves show, a trend that is consistent with constructed concrete nonlinear creep damage evolution, and the fitting correlation coefficient is more than 0.96, as shown in Table 1 and Figure 4. The fitting results show that the concrete instantaneous elastic deformation after loading can be correctly described by the constitutive model. Meanwhile, the initial creep of stage 1 and the steady creep of stage 2 can also be correctly expressed.

### 4.2. Experimental Research on Concrete Creep Under Different Stresses

**Figure 5:**
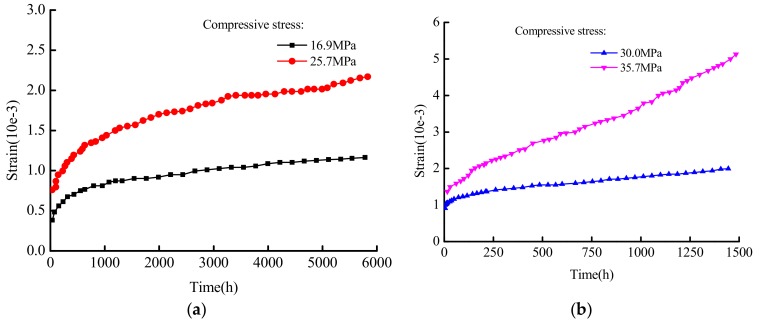
The curve of concrete creep test under different stress. (**a**) 16.9 MPa and 25.7 MPa; (**b**) 30 MPa and 35.7 MPa.

Compressive nonlinear creep and residual strain tests on C40 concrete specimens under different stresses were carried out by Can Tang et al. [41]. The concrete specimens were prisms with the size of 100 mm × l00 mm × 400 mm. The stress was controlled at 12.1 MPa, 16.9 MPa, 20.7 MPa, 25.7 MPa, 30.0 MPa, and 35.7 MPa. The cubic compressive strength and axial compressive strength was 50.4 MPa and 40.9 MPa, respectively, under a 28-day standard. The nonlinear creep test curve is shown in Figure 5.

**Figure 6:**
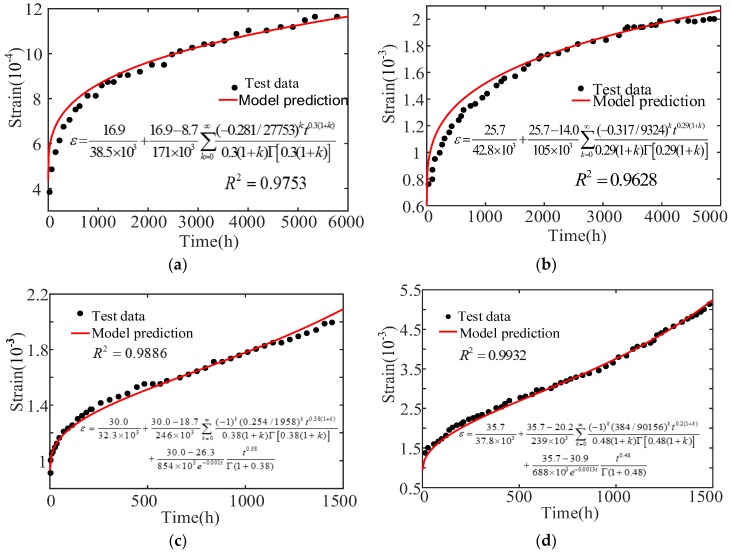
Fitting curves of test curves and built models. (**a**) *σ* = 16.9 MPa; (**b**) *σ* = 25.7 MPa; (**c**) *σ* = 30.0 MPa; (**d**) *σ* = 35.7 MPa.

Under stress of 16.9 MPa and 25.7 MPa, the instantaneous creep occurred first and then the isometric creep, and the creep was velocity first increased and then decreased, as shown in Figure 5a. The creep process includes elastic strain, deceleration creep and isovelocity creep. The experimental curve characteristics can be described using the elastomer and viscoelastic body in the model. At this time, the parameters $E_{0}$,$E_{1}$, $\sigma_{S1}$ and $\eta_{1}$, and the fractional order n are selected according to the curve characteristics. Under stress levels of 30.0 MPa and 35.7 MPa, as shown in Figure 5b, the concrete specimen creep process consists of instantaneous creep, isovelocity creep, and accelerated creep, and the applied stress is greater than $\sigma_{S2}$. The experimental curve characteristics can be described using the elastomers, viscoelastics, and viscous bodies in the model. Now, the parameters $E_{0}$, $E_{1}$, $\sigma_{S1}$, $\eta_{1}$, $\sigma_{S2}$, and $\eta_{2}$ need to be solved, and the fractional order n can be selected according to the curve characteristics. The numerical fitting analysis of the test results are carried out using the conducted model. The calculation model parameters are shown in Table 2. The fitting results are shown in Figure 6.

Can Tang et al.’s [41] test curves show the same trend as with the concrete nonlinear creep damage evolution, and the fitting correlation coefficient is 0.96, which has a good correlation, as shown in Table 2 and Figure 6. The fractional order creep model represents the deceleration and isovelocity creep of the concrete, and it also express the accelerated creep stage by comparison. It has good consistency for the entire concrete creep process. In summary, the concrete creep damage evolution rule, under high and different levels of stress, can be well reflected by the constructed concrete creep damage constitutive model proposed in this paper. It is reasonable and feasible to describe the three stages of concrete creep deceleration creep, constant, and accelerated creep and the model has certain application and reference value. Meanwhile, the application of fractional calculus theory to improve traditional elements is also further illustrated, as this theory has become one of the important means to study the concrete creep nonlinearity. The research results are consistent with Zhilei He [42] and HW Zhou [36,43].

## 5. Parameter Sensitivity Analysis of Concrete Nonlinear Creep Damage Constitutive Model

Parameters *σ*, *n*, and α are the main factors affecting the concrete creep in the concrete creep damage constitutive model; these parameters are obtained by analyzing the experimental data curve and the theoretical curve. Therefore, the sensitivity analysis of the model parameters will be carried out by the control variable method in this section to study the influence of the above model parameters on concrete creep. The model parameters are as follows: σ=30·MPa, $E_{0}=32.3GPa$, $E_{1}=254GPa$, $\sigma_{S1}=18.7MPa$, $\eta_{1}{}^{n}=246GPa\cdot h$, $\sigma_{S2}=26.3MPa$, $\eta_{2}{}^{n}=854GPa\cdot h$, α=0.001 and n=0.38. The concrete compression creep and residual strain experimental data of Can Tang are selected, and the stress level is 30.0 MPa. 

### 5.1. Effect of Stress Level σ

**Figure 7:**
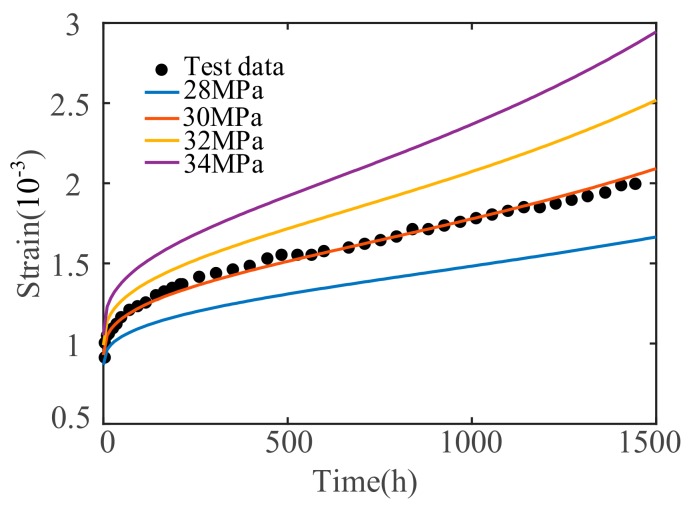
Sensitivity analysis under different stress σ.

Parameters $E_{0}=32.3GPa$, $E_{1}=254GPa$, $\sigma_{S1}=18.7MPa$, $\eta_{1}{}^{n}=246GPa\cdot h$, $\sigma_{S2}=26.3MPa$, $\eta_{2}{}^{n}=854GPa\cdot h$, α=0.001 and n=0.38 are selected and brought into Equation (23). The stress level varies: 28 MPa, 30 MPa, 32 MPa, and 34 MPa. The creep test results at different stress levels are shown in Figure 7.

The concrete creep deformation is greatly affected by the stress level, and the creep deformation will be increased as the stress level increases. However, stress has no obvious effect on the creep velocity. The higher the stress level, the greater the instantaneous deformation in the elastic stage, and the shorter the time required to enter steady state creep at a constant velocity. In addition, the form of the concrete creep curves is gradually changed to the exponential type as the stress level increased. When the stress level exceeds a certain level, a sudden change will occur. At this point, micro-cracks will occur, eventually causing macro-crack penetration failure. This is consistent with Narintsoa Ranaivomanana et al.’s test [40] of how high stress can accelerate the creep process.

### 5.2. The Influence of Fractional Order n

**Figure 8:**
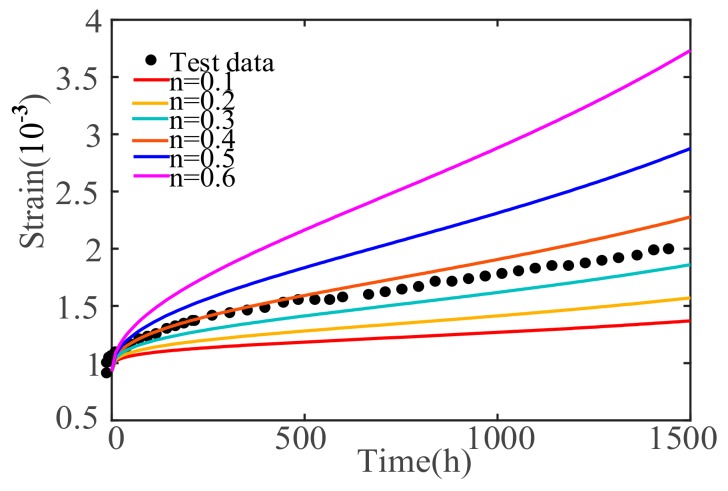
Sensitivity analysis under different orders of *n.*

In order to analyze the sensitivity of the fractional order, the control variable method is used to ensure that other parameters remain unchanged. The fractional order is gradually increased from 0.1 to 0.6, and a series of creep curves with different orders are obtained, as shown in Figure 8.

From Figure 8, we can see that the duration of instantaneous creep is shortened with the increase of the fractional order and the duration of deceleration and isovelocity creep, and of the concrete entering the stage of accelerated creep, is correspondingly also shortened. The concrete creep deformation is increased gradually as the fractional order increases. In addition, the concrete creep velocity is affected by the fractional order number; that is, the higher the fractional order, the greater the creep rate at each stage.

### 5.3. Effect of Material Parameter α

**Figure 9:**
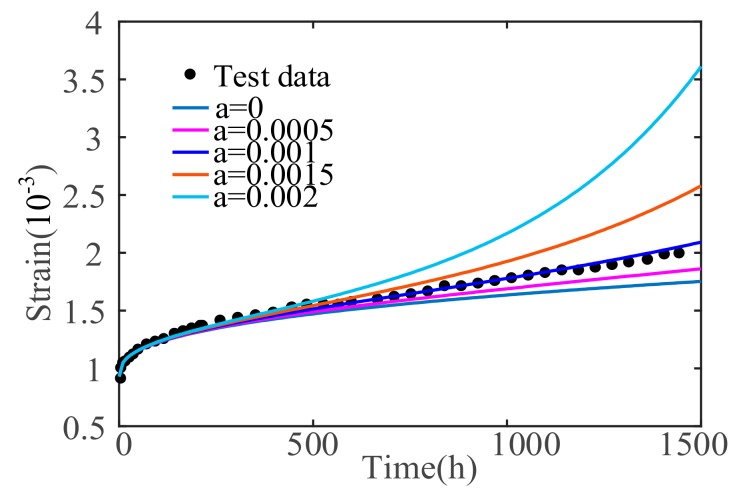
Sensitivity analysis under different material parameters *α*.

Considering the change in the damage variable in a viscoplastic body, the control variable method is used to obtain the creep curves under different α, by changing the value of *α*, as shown in Figure 9. As material parameter *α* increases, the sooner the accelerated creep phase begins, and the greater the creep velocity will be. The power index is the main factor that influences creep velocity. The larger the power exponential value, the more prone the concrete will be to accelerated creep.

The classical rheological model and the Fractional order theory are introduced to inform the concrete nonlinear creep damage constitutive model after the results of the above research. The conducted constitutive model is a viscoelastic-plastic model. 

It is assumed that the damage in the deceleration creep stage and in the stable creep stage is neglected, and only the damage in the accelerated creep stage is considered. Also, the accelerated creep rate conforms with changes in the exponential function charge in the indoor concrete creep tests. In practice, the above two assumptions may not be agreed with each other. Therefore, the concrete fractional nonlinear creep damage constitutive model derived from this assumption differs from the actual situation. However, the concrete specimen damage data contributes little to the constitutive equation in either the initial stage or the stable creep stage in the actual uniaxial compression creep tests. The constructed concrete creep damage model is more able to meet the needs of actual working conditions and tests that consider accelerated creep stage damage as the main research object. In addition, the constructed concrete creep damage model parameters have specific physical significance in this paper, and the entire concrete creep process in practical engineering can be reflected and described by the proposed model, which has certain engineering practical and reference values.

## 6. Conclusions

To better describe the concrete non-linear creep damage evolution rule, and better solve the concrete creep in concrete engineering, Fractional Calculus Theory is introduced to inform a concrete nonlinear creep damage constitutive model. The conducted constitutive model is verified and analyzed for sensitivity by means of the concrete creep test. The following conclusions may be drawn:

1. The Visco-elasticity Plasticity Rheological Theory, the Riemann Liouville Theory and the Combined Model Theory are quoted to propose the stress-strain relationship expressions of the elastomer body, the visco-elasticity plasticity body, and the viscoplastic body. The nonlinear creep damage evolution equation of the concrete fractional order is conducted with consideration of damage evolution, and the parameter specific calculation method in the constructed model is given.
2. The constructed concrete creep damage constitutive model in this paper is validated in high stress and different stress situations that involve uniaxial compression creep; data from the residual strain tests of Narintsoa Ranaivomanana et al. [40] and Can Tang [41] is considered. The results show that the conducted model can accurately describe the entire concrete creep process, and that it could be used as a reference for concrete non-linear creep.
3. To determine the influence degree of the model parameters, the sensitivity analysis of the main influence parameters in the concrete creep damage constitutive model, including *σ*, material parameter *α*, and fractional order *n*, are analyzed. The results show that *σ* and *n* are positively correlated with the concrete creep deformation and rate. The creep deformation and rate will increase as *σ* and *n* increase. *α* is related to the time at which the concrete enters the accelerated creep stage, and the larger the value of material parameter *α*, the earlier the concrete will enter the accelerated creep stage.

## Figures and Tables

**Table 1:** Parameter table of concrete creep model.

Stress/MPa	$E_{0}/\mathrm{GPa}$	$\sigma_{S1}/\mathrm{MPa}$	$\eta_{1}{}^{n}/\mathrm{GPa}\text{·}h$	$E_{1}/\mathrm{GPa}$	$\sigma_{S2}/\mathrm{MPa}$	$\eta_{2}{}^{n}/\mathrm{GPa}\text{·}h$	*α*/*h*^-*i*^	*n*
20.9	45.2	13.5	235	263	–	–	–	0.31
33.9	40.1	24.0	191	332	–	–	–	0.36

**Table 2:** Parameter table of concrete creep model.

Stress/MPa	$E_{0}/\mathrm{GPa}$	$\sigma_{S1}/\mathrm{MPa}$	$\eta_{1}{}^{n}/\mathrm{GPa}\text{·}h$	$E_{1}/\mathrm{GPa}$	$\sigma_{S2}/\mathrm{MPa}$	$\eta_{2}{}^{n}/\mathrm{GPa}\text{·}h$	*α*/*h*^-*i*^	*n*
16.9	38.5	8.7	171	281	–	–	–	0.3
25.7	42.8	14.0	105	317	–	–	–	0.29
30.0	32.3	18.7	246	254	26.3	854	0.001	0.38
35.7	37.8	20.2	239	384	30.9	688	0.0013	0.48
